# Supplementary material for: Mixed-Precision Deep Learning Based on Computational Memory
Source: Front Neurosci. 2020 May 12;14:406. doi: 10.3389/fnins.2020.00406 (PMC7235420; doi:10.3389/fnins.2020.00406)
Supplement: Supplementary file 1 [file Data_Sheet_1.pdf]

Mixed-precision deep learning based on computational memory – Supplementary Material

S. R. Nandakumar,[1] Manuel Le Gallo,[1, a)] Christophe Piveteau,[1,2] Vinay Joshi,[1,3] Giovanni Mariani,[1] Irem Boybat,[1,4] Geethan Karunaratne,[1,2] Riduan Khaddam-Aljameh,[1,2] Urs Egger,[1] Anastasios Petropoulos,[1,5] Theodore Antonakopoulos,[5] Bipin Rajendran,[3, b)] Abu Sebastian,[1, c)] and Evangelos Eleftheriou[1]

[1)] *IBM Research - Zurich, 8803 Rüschlikon, Switzerland*
[2)] *ETH Zurich, 8092 Zurich, Switzerland*
[3)] *King's College London, London WC2R 2LS, United Kingdom*
[4)] *Ecole Polytechnique Federale de Lausanne (EPFL), 1015 Lausanne, Switzerland*
[5)] *University of Patras, 26504 Rio Achaia, Greece*

(Dated: 22 April 2020)

[a)] Electronic mail: anu@zurich.ibm.com
[b)] Electronic mail: bipin.rajendran@kcl.ac.uk
[c)] Electronic mail: ase@zurich.ibm.com

SUPPLEMENTARY FIGURE 1: TRAINING EXPERIMENT USING NON-DIFFERENTIAL PHASE-CHANGE MEMORY SYNAPSE

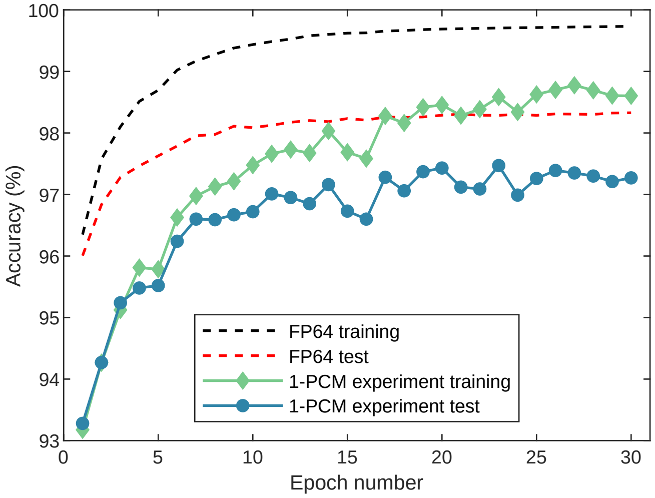

Supplementary Fig. 1. Training and test accuracies from the mixed-precision computational memory architecture (MCA) training experiment for MNIST digit classification using the network in Fig. 3**a** in the main article, where weight increments and decrements are implemented by applying SET and RESET pulses to the same phase-change memory (PCM) device per synapse. Irrespective of the conductance change asymmetry, the training achieves a maximum training accuracy of 98.77% and a maximum test accuracy of 97.47% in 30 epochs, within 1% of that obtained in corresponding high precision (FP64) training. FP64 training delivers training and test accuracies of 99.73% and 98.33%, respectively. This experiment demonstrates the ability of the MCA to compensate for the conductance update asymmetry often observed in the nano-scale memristive devices. See Methods for details on the training experiment.

SUPPLEMENTARY NOTE 1: STATISTICAL MODEL OF THE PHASE-CHANGE MEMORY DEVICE

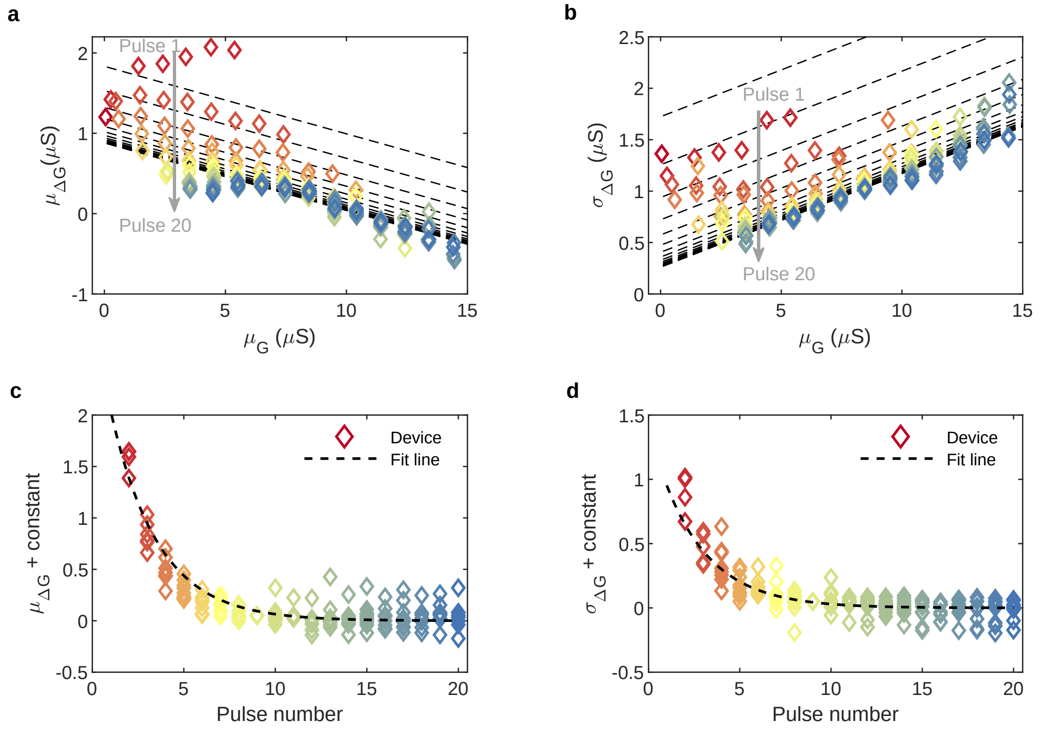

Supplementary Fig. 2. State dependence of conductance change observed in the PCM and its model approximation. (a) The mean of the conductance change versus mean initial conductance (b) The standard deviation of the conductance change versus mean initial conductance. (c), (d) The same data points as in (a) and (b) are plotted as a function of pulse number. A scalar constant per curve was added to each of these curves to remove the initial conductance dependency. The resulting relation of $\mu_{\Delta G}$ and $\sigma_{\Delta G}$ with pulse number are approximated as exponentially decaying functions with a decay constant, $\alpha = 2.6$.

A statistical model for phase-change memory (PCM) device programming was developed to predict the next state of the device given the current state and the programming pulse characteristics. For this, we used the device data from Fig. 2a in the main article that show the statistics of cumulative conductance evolution from 10,000 devices upon applying 20 SET programming pulses of 90 μA amplitude and 50 ns width. In between the application of each SET pulse, the devices were read 50 times. The data from the 50th read after each programming pulse, corresponding to a time delay of $t_0 = 38.6$ s on average from programming, were used to develop the model. The state-dependent behavior of the conductance change due to each programming pulse is analyzed as shown in Supplementary Fig. 2. The initial conductance distribution before each programming pulse is divided into bins of width 1 μS. The mean ($\mu_{\Delta G}$) and standard deviation ($\sigma_{\Delta G}$) of the conductance change ΔG (difference between device conductance before and after the application of the SET pulse) are plotted with respect to the mean initial conductance (μ_G) of each bin. In Supplementary Fig. 2, the color transition from red to blue corresponds to increasing programming pulse numbers from 1 to 20. The relations $\mu_{\Delta G}$ vs μ_G and $\sigma_{\Delta G}$ vs μ_G could be approximated using straight lines whose y-intercept decays exponentially with the number of programming pulses (Supplementary Fig. 2 c, d). The dependence of the conductance change upon the initial state and the pulse number can be captured using the following relations:

$$\mu_{\Delta G} = m_1 \mu_G + (c_1 + A_1 e^{-p/\alpha}) \tag{1}$$

$$\sigma_{\Delta G} = m_2 \mu_G + (c_2 + A_2 e^{-p/\alpha}) \tag{2}$$

The factor $e^{-p/\alpha} (= \prod_1^p e^{-1/\alpha})$ captures the effect of applying p programming pulses to the device. The values of the parameters used for the model are listed in Supplementary Table I. In order to simulate a single device programming event using this model, μ_G in the equations is replaced by the corresponding individual device conductance value. Equations (1) and (2) can be used to estimate the device conductance after time t_0 from a SET programming pulse. However, the device conductance at other points in time will be different due to conductance drift and read noise. The average conductance evolution over time during

the 20 SET pulses programming sequence is shown in Supplementary Fig. 3a. We observe that the conductance drift re-initiates after the application of each SET pulse. The conductance drift in PCM devices can be modeled with the following equation:

$$G(t) = G(t_0)\left(\frac{t}{t_0}\right)^{-\nu} \tag{3}$$

which relates the conductance at any time t elapsed after a programming event to the conductance $G(t_0)$ measured at a time t_0 after programming. The device measurement data from the consecutive reads after each programming pulse is used to estimate the conductance drift. The 50 reads from the programmed states along with an immediate read approximately after 100 ns from the programming pulse, averaged over 10,000 devices, are fitted with Equation 3 (Fig. 2c in the main article). The extracted drift-coefficient as a function of the mean conductance state at the 50th read is plotted in Supplementary Fig. 3b. The average drift coefficient value is 0.04.

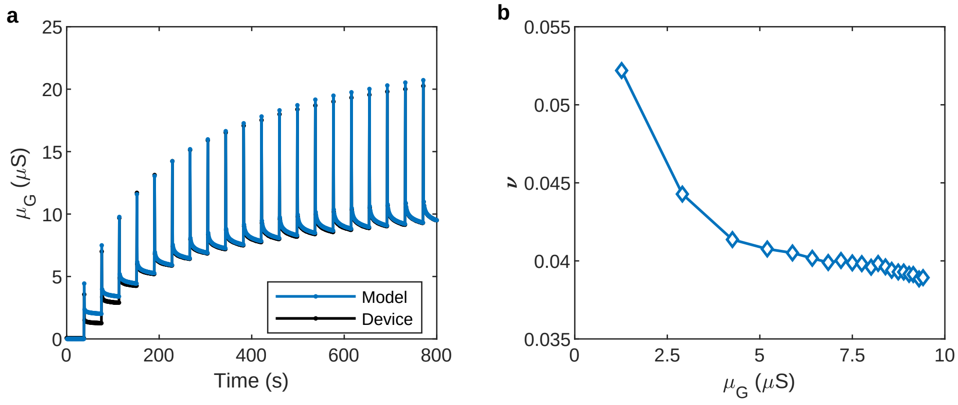

Supplementary Fig. 3. (a) Average conductance evolution of 10,000 PCM devices when subjected to 20 SET pulses with 50 interleaved reads. The peak points represent the first conductance read approximately 100 ns after each programming pulse. The overlapping model response demonstrates its agreement with the experiment in capturing drift behavior. Note that the drift is reinitiated after each SET programming pulse. (b) The drift coefficients extracted from the temporal response of the PCM devices in (a) (see also Fig. 2c in the main article) based on Equation 3.

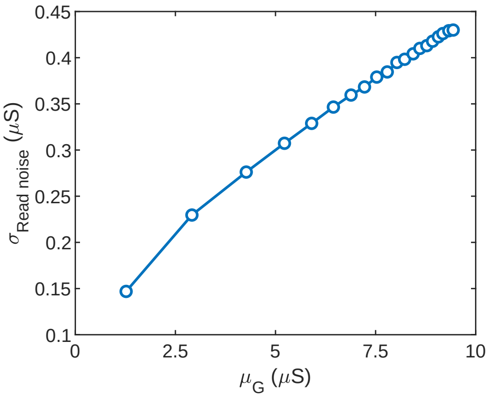

Supplementary Fig. 4. The standard deviation of the read noise extracted from the device array as a function of average current conductance state.

The standard deviation of the read noise was estimated using the last 10 reads out of the 50 reads during which the impact of conductance drift is minimum. The read noise is assumed to be a zero-mean Gaussian distributed additive noise over the drifted conductance. The measured standard deviation of this read noise as a function of the average conductance is shown in Supplementary Fig. 4. The read noise dependence on the average conductance can be modeled as a straight line of the form, $y = m_3x + c_3$, and the parameters m_3 and c_3 are presented in Supplementary Table I. In the training simulations, the read noise is modeled as zero mean Gaussian noise with a standard deviation of 0.4 μS.

Supplementary Table I. The PCM model parameters

Symbol	Value	Symbol	Value	Symbol	Value
m_1	-0.084	c_1	0.880	A_1	1.40
m_2	0.091	c_2	0.260	A_2	2.15
α	2.6	t_0	38.6 s	ν	0.04
m_3	0.03	c_3	0.13		

SUPPLEMENTARY NOTE 2: ON-CHIP OHMIC MULTIPLICATION EXPERIMENT

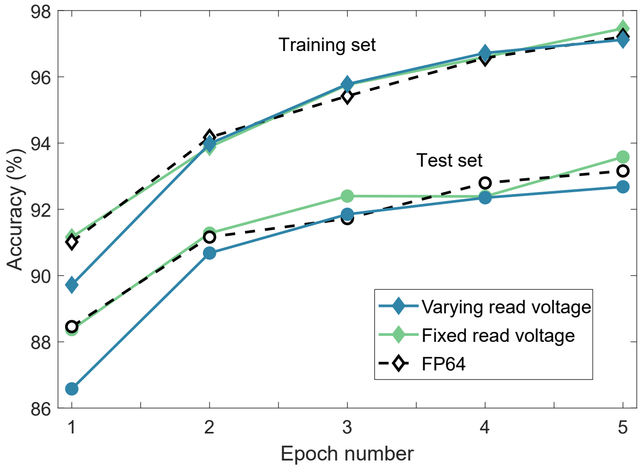

Supplementary Fig. 5. Training experiment using varying read voltage and fixed read voltage. Classification accuracies are based on the 5000 training images and 10,000 test set images. The experiment using varying read voltages read the currents from all the devices, for all training and test images. Fixed read voltage experiment was performed by reading a subset of devices in a round robin fashion. Accuracies from the floating-point reference (FP64) are also shown.

In the experiments in the main manuscript, we used a constant read voltage and measured the resulting current/conductance from the PCM array for the propagation stages. This is indeed in-memory multiplication and note that there is no loss of generality especially if a pulse-width modulation scheme is used to code the input to the array. But one could argue how that compares with a scenario where the read voltage is varied.

To address this, we performed an additional training experiment. In this experiment, for each training image, the neuron activations and error vectors were mapped to read voltages between 0 and 0.3V and the resulting currents from the respective PCM devices were read from the hardware array implementing Ohm's law on-chip. This validation experiment was limited to 5 epochs of training using 5000 images and test accuracies are reported based on 10,000 images. All devices of the array were read with varying voltages for every single matrix-vector multiplication performed during both training and testing. This training experiment reaches the floating-point classification accuracy irrespective of the device non-linearities (Supplementary Fig. 5). A second simplified experiment was conducted, where a fixed read voltage of 0.3 V was used and only a subset of all the devices were read. We read all the devices from the second layer, and 785 pairs of devices from the first layer after each training example involving a conductance update event in the hardware array. The 785 pairs are chosen in a round robin fashion such that after 250 device update events all the devices in the first layer are updated at least once in the training simulator. It can be seen that the performance of this second experiment compares well with the experiment where the read voltages are varied. Hence, the large scale training experiment of the manuscript using the full dataset followed the second approach.

SUPPLEMENTARY NOTE 3: PCM MODEL BASED VALIDATION OF TRAINING EXPERIMENT

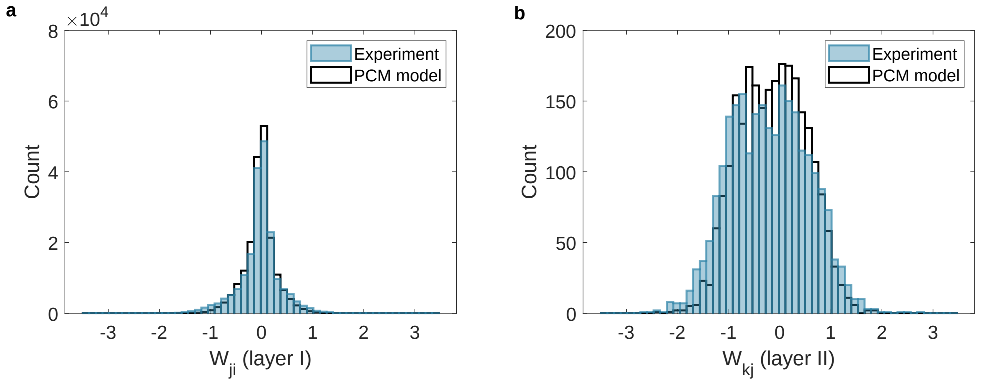

Supplementary Fig. 6. Synaptic weight distribution at the end of training from the two layer network used for MNIST handwritten digit classification. The PCM model conductance distribution in the two weight layers (layer 1 in (a) and layer 2 in (b)) at the end of 30 epochs of training matches that from the experiment.

We used the PCM model to validate the experimental training behavior of the mixed-precision computational memory architecture (MCA) for the classification of MNIST handwritten images. Supplementary Fig. 6 shows that the weight distribution at the end of 30 epochs of training experiment matches with that from the simulation. It indicates that the model is able to capture the overall device programming dynamics in the training scenario which involves many conductance update events having varying intermediate delays and occasional RESETs.

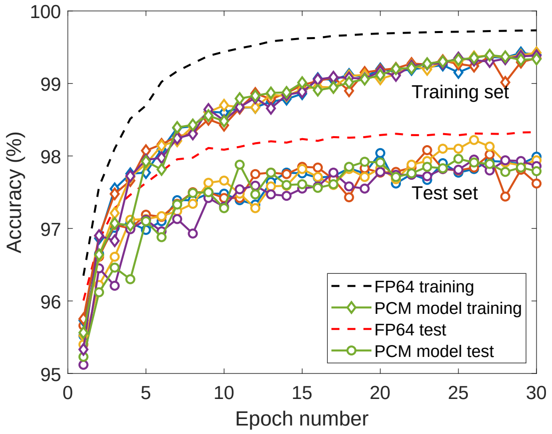

Supplementary Fig. 7. Classification performance when the two layer network for MNIST digit recognition is trained with the stochastic PCM model using 5 different random seeds.

Simulations of the training using multiple random seeds for the stochastic device programming in the model are shown in Supplementary Fig. 7. Here, the maximum test accuracy reaches within 0.1% from that of FP64-based training even though the training time is slightly longer compared to the FP64 implementation. This can be attributed to the stochastic conductance update of the PCM devices. This behavior could be eventually mitigated by using multiple PCM devices per synapse, which has been shown to reduce the overall update stochasticity[1].

SUPPLEMENTARY NOTE 4: IMPACT OF DEVICE NON-IDEALITIES

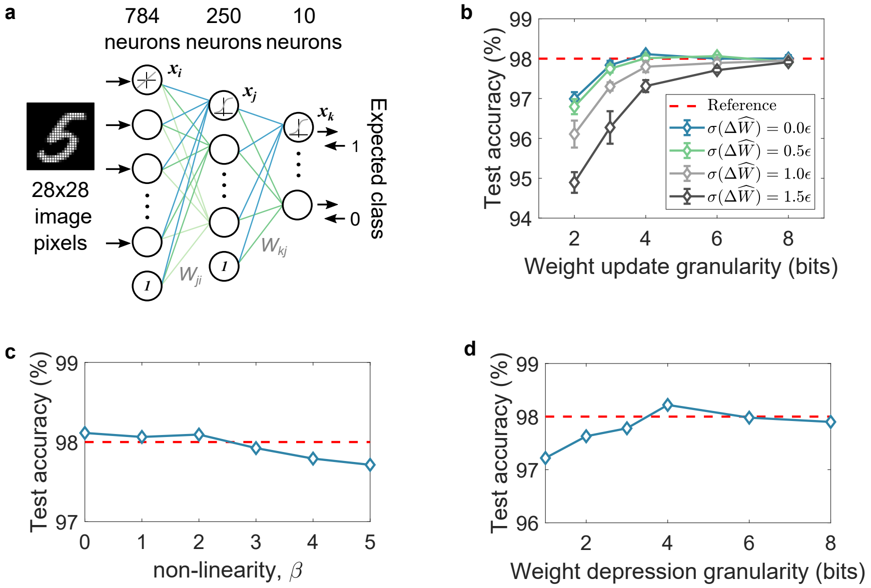

Supplementary Fig. 8. (a) The two-layer perceptron used to evaluate the MCA. The network is trained using 60,000 MNIST training images. After 10 epochs of training in 64-bit precision the network achieves 98% accuracy in classifying 10,000 test images (Reference). (b) Effect of weight update precision and stochasticity. The weights are realized using linear device models with symmetric potentiation and depression. The weight values are in the range [-1, 1] and the weight update size is determined as $\varepsilon = 2/(2^n - 2)$ for n-bit update granularity. The standard deviation of weight update is varied as a multiple of ε. (c) Test accuracy versus varying amount of update non-linearity. $\beta = 0$ corresponds to linear update with 4-bit precision and $\beta = 5$ corresponds to nearly an abrupt transition between the weight range boundaries. (d) Effect of update asymmetry. The weight depression resolution is varied while the potentiation resolution is kept at 8-bit.

In a previous work, we evaluated the training efficacy of the architecture using simple device models to study the effects of various non-idealities independently[2] (Supplementary Fig. 8). A two layer perceptron is trained to classify MNIST images. Under conventional software training with floating-point precision, the network attains 98% test accuracy after 10 epochs of training. If the computational memory devices are realized using linear models with symmetric potentiation and depression, a test accuracy of approximately 97% can be maintained when trained using MCA with merely three weight levels {-1, 0, 1} (Supplementary Fig. 8 b). We further evaluated the effect of weight update noise. With a high programming noise standard deviation of 1.5 times the average weight update size, the network still maintained above 94% test accuracy. Using suitable weight update thresholds in the MCA, the network also managed to have less than 1% test accuracy drop in highly non-linear and asymmetric weight update training (Supplementary Fig. 8 c, d).

Using the PCM model developed in this work (see Supplementary Note 1), we further evaluated the effect of PCM non-idealities on the network accuracy. The PCM model also incorporates the conductance drift. Since the training algorithm is observing the error introduced by the drift during training, it is getting compensated during further training, and creates a slightly drift resilient solution. We performed an additional training simulation using the model to determine the source of 0.6% accuracy drop in the MNIST training experiment. For that we modified the model such that it has conductance drift as observed from our devices, however, the programming stochasticity is scaled to be 50% of that from device (Supplementary Fig. 9a). The training response is shown in Supplementary Fig. 9b. The training achieves high-precision equivalent accuracy. Hence, we concluded that the primary source of performance drop during training in our experiment is the programming noise.

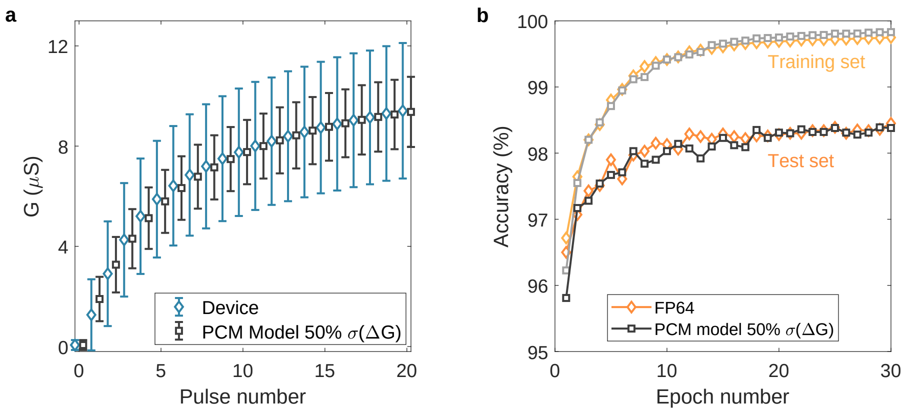

Supplementary Fig. 9. (a) PCM model response with 50% conductance update variability. Error bars correspond to one standard deviation. (b) Simulated classification accuracy on the training and test set using the PCM model with 50% update variability. High-precision (FP64) training results are shown for reference.

SUPPLEMENTARY NOTE 5: INFLUENCE OF THE REFRESH METHODOLOGY

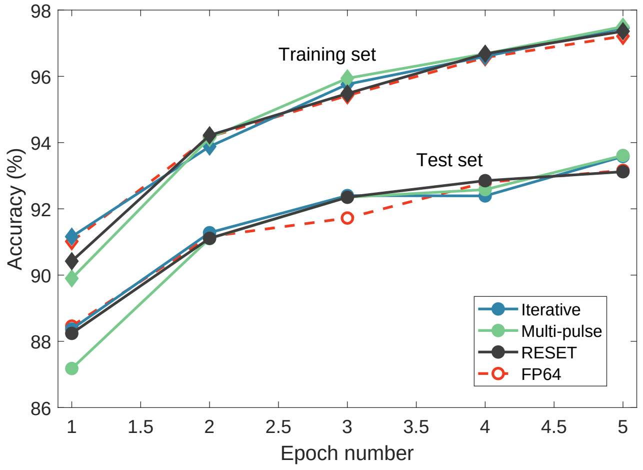

Supplementary Fig. 10. A comparative study on the impact of different refresh schemes on training and test accuracies using on-chip training experiments. The training experiments were performed with 5,000 training images and 10,000 test images from the MNIST dataset.

When the neural network weights are implemented as the difference of two PCM devices ($W = \beta(G_p - G_n)$), the weight increment (decrement) is achieved by a SET pulse to the G_p (G_n) device. This leads to a monotonic increase in the conductance and the conductance of devices receiving sufficiently large number of programming pulses will eventually saturate. This necessitates periodic detection of saturated devices and their reprogramming. In our training experiment, the weight refresh is implemented as follows. After every 100 training examples, all the device conductance pairs are read. The conductance pair is checked if one of them is above $8\,\mu$S and if the difference between them is less than $6\,\mu$S. When this condition is met, a weight refresh operation is performed. An ideal weight refresh operation will RESET both G_n and G_p and their original conductance difference $G_p - G_n$ is reprogrammed to one of these devices based on the sign of the difference. However, PCM programming is a stochastic process. While the RESET could be performed using a single pulse of sufficient amplitude, the conductance reprogramming to a reasonable accuracy could take many write-read-verify cycles and hence could be expensive. Here, we experimentally verify the impact of various simpler conductance reprogramming methods.

We repeated the MNIST training experiment in hardware using the array of PCM devices with three different reprogramming methods. In the first case, the conductance reprogramming used the iterative method to accurately reprogram the difference[3]. In the second case, identical to the procedure used in the training experiment of the main manuscript, the conductance difference was divided by the average conductance change to determine the number of programming pulses to approximately reprogram the difference. Then, we blindly applied the programming pulses without a read-verify operation. In the third case, we completely avoided the reprogramming, and the weight refresh operation involved only a RESET operation to zero conductance value. A subset of the original training dataset was used to validate the experiment using these refresh schemes. We trained the network using 5,000 images for 5 epochs, and tested using 10,000 test images. The resulting experimental training and test accuracies are plotted in Supplementary Fig. 10. The corresponding high-precision training results are also shown for reference. The classification performance from the three schemes almost overlap. Also, the results from these training experiments overlap the accuracy values obtained from the corresponding high-precision training.

The results indicate that the weight refresh operation could be as simple as blindly resetting devices in the array. This RESET based weight refresh scheme brings the devices out of saturation and allows further updates. The downside is that the number of programming pulses necessary for the weight update increases as we go from (most accurate) iterative refresh to multi-pulse refresh, and to RESET based refresh (least accurate). Investigating the trade-offs between different refresh schemes at the system-level and developing pipelining schemes that efficiently accommodate refresh in the MCA will be an important subject of future work.

SUPPLEMENTARY NOTE 6: THE SIMULATOR

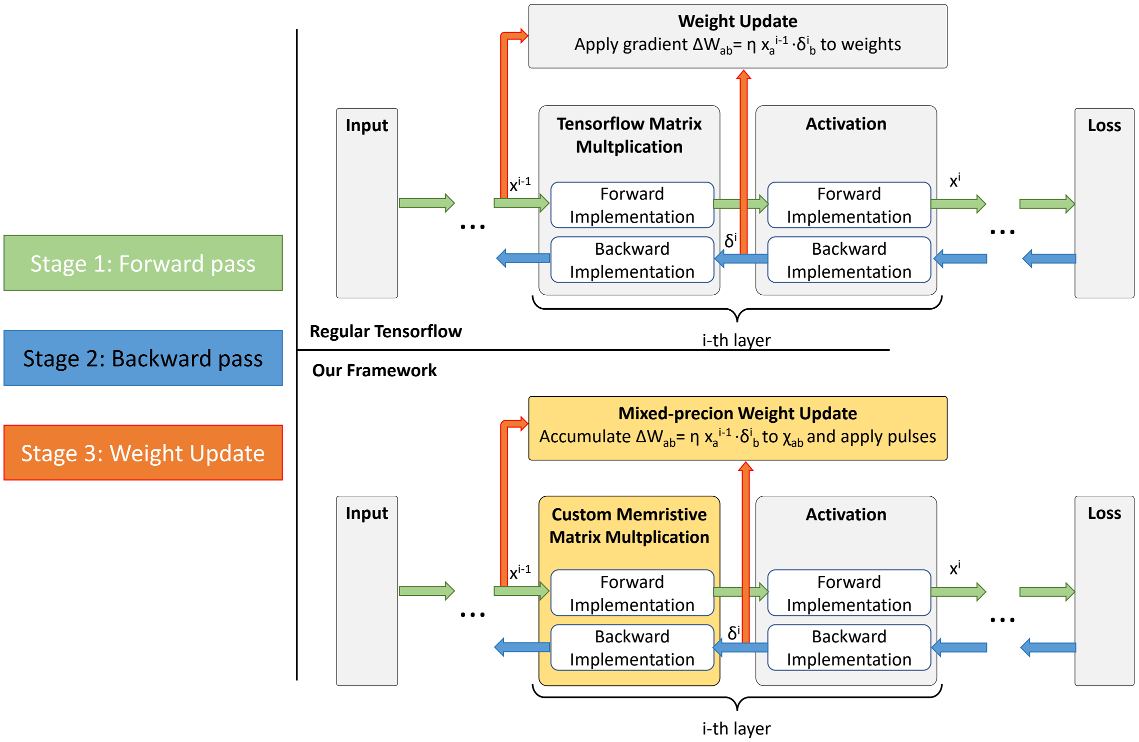

Supplementary Fig. 11. Schematic representation of the inner workings of a fully-connected layer in our simulation framework. The regular TensorFlow matrix multiplication operation is replaced with a custom operation which takes into account the quantization effects and device behavior. A similar structure holds for the convolution layers once the convolution kernels are re-arranged to form a matrix (see Supplementary Fig. 13).

In this note, we discuss how the model of the PCM based computational memory, which captures the programming and temporal evolution behavior from the actual devices, is integrated with the TensorFlow deep learning framework, and is used to test the efficacy of the MCA for deeper and more complex neural network training problems.

Training of deep neural network is of significant mathematical complexity and has high computational resource requirement. For this reason, most modern deep learning codes are written on top of already existing deep learning frameworks that significantly simplify the handling of larger and newer models. These frameworks typically give access to a wide array of neural network building blocks which can be chained together to construct complex networks with ease. They also provide the gradient computation using automatic differentiation. Furthermore, these frameworks make it possible to run the model on a General Purpose Graphical Processing Unit (GPGPU) - which has become a necessity for training deep networks. Hence, we decided to implement our simulator as an extension to Google's TensorFlow deep learning framework. The integration into TensorFlow also allows us to make use of its large library of pre-existing algorithms, making it is rather straightforward to integrate techniques such as dropout or pooling. Furthermore, it implies that regular TensorFlow codes can be easily converted to perform corresponding mixed-precision training simulations.

As represented in Supplementary Fig. 11, custom TensorFlow operations were implemented that take into account the various attributes of PCM devices such as conductance range, read noise, and conductance drift as well as the characteristics of the data converters. During forward and backward data propagation, some of the system non-idealities (read noise and quantization due to converters) have to be recomputed independently. Since TensorFlow's automatic differentiation algorithm cannot re-evaluate the non-idealities separately for forward and backward pass, we implemented custom gradient backpropagation routines for the MCA operations. The read noise of PCM devices is incorporated by adding an additive noise term to the weight matrices each time they are accessed during the forward and backward propagation stages. This random noise is sampled independently every time the weight matrix is invoked. The distribution of the read noise is chosen to be Gaussian with zero mean and 0.4 μS standard deviation. Furthermore, we have replaced the synaptic weight update routines with custom ones that implement the high-precision accumulation and conductance updates using pulse programming based on the statistical model described in Supplementary Note 1 and Section 2 of the main manuscript. The neural network weights in the simulator are implemented

using two PCM devices in differential configuration. The simulator also captures the weight refresh operation similar to the on-chip training experiment, with a suitable refresh condition and refresh frequency.

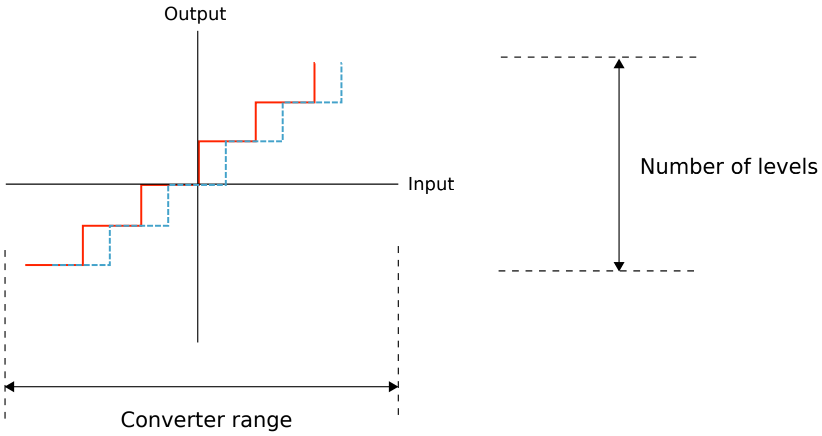

Supplementary Fig. 12. Quantization function response for analog-digital converters. The quantization range and the number of quantization levels can be individually set. The function is chosen such that there is no level transition at $x = 0$ as seen in the blue curve as opposed to the red curve.

The simulator captures the quantization effects arising from the analog to digital (A-to-D) and digital to analog (D-to-A) data converters at the crossbar array periphery with high fidelity. The quantization function $q(x)$ taking the input x as its argument depends on two parameters: the number of quantization steps (chosen as 2^8 corresponding to 8-bit converters) and the quantization range $[q_{min}, q_{max}]$ that determines the dynamic range of the converter. From our experience, the quantization function should not have a level transition at $x = 0$ (i.e. as seen with the blue curve in Supplementary Fig. 12 as opposed to the red curve), as this leads to a severe performance degradation, due to near-zero activations and gradients being mapped to a value which is much larger in magnitude. Considering that in most cases one wants the range to be symmetric ($q_{min} = -q_{max}$), we chose the quantization function to be:

$$q(x) = clip\left(\frac{q_{max} - q_{min}}{2^n} \cdot round\left(\frac{2^n}{q_{max} - q_{min}}(x - q_{min})\right) + q_{min}\right) \quad (4)$$

where $clip(x)$ clips the value of x to $[q_{min}, q_{max}]$ and $round(x)$ rounds x to the nearest integer value.

Since the magnitude of the backpropagated gradients changes over training examples, they are normalized before feeding into the crossbar array. The resulting output vector from the array is scaled back using the same normalization factor. The full matrix-vector multiplication $W \cdot x$ with a matrix W and an input vector x is therefore simulated in the following manner:

$$q_{A-to-D}\left(W \cdot q_{D-to-A}\left(\frac{x}{||x||}\right)\right)||x|| \quad (5)$$

where $q_{D-to-A}()$ and $q_{A-to-D}()$ are the quantization functions for the corresponding data converters.

In order to simulate the conductance drift behavior of PCM devices, the simulation framework also incorporates a notion of time. A global timer is incremented after every mini-batch. The time per batch is assumed to be 1 ms for all the networks simulated. For every synapse, we store the time at which it was last updated. The conductance states are represented by storing the $G(t_0)$ predicted by the model, with t_0 being a model parameter representing a fixed time from the last update for each device. At every read operation, the current conductance values are computed using equation (3) depending on the time of last update, the global timer, and $G(t_0)$. The timer is not updated inside a mini-batch for implementation simplicity and to save simulation time. Indeed, assuming the same weight matrix for all the examples in a batch allows to utilize the faster batch-processing provided by GPUs.

The actual time scale of data propagation through the crossbar arrays might vary depending on the hardware implementation. However, we can observe that changing the time scale of the network operation by a constant λ leads to all conductance values to be adjusted by the same coefficient: from equation (3), $G(\lambda t) = \lambda^{-\nu} G(t)$. Therefore, changing the time scale by the factor λ has the equivalent effect of scaling all conductance-to-weight mapping ratio by $\lambda^{-\nu}$. Hence, in the eventual hardware implementation, the deviations in the actual computation time per crossbar array could be compensated by a scaling factor at the peripheral circuits, to maintain similar training performance.

SUPPLEMENTARY NOTE 7: NETWORK IMPLEMENTATION IN MCA AND TRAINING

Convolutional Neural Network

The details on how the convolution layers of a convolutional neural network (CNN) are mapped to computational memory in the mixed-precision architecture for training are shown in Supplementary Fig. 13. We follow the same approach as that of Shafiee et al.[4] and Gokmen et al.[5]. This rearrangement of convolution filters in the form of a matrix, suitable for crossbar array, allows the forward and backward data propagation to be implemented in the form of matrix-vector multiplication analogous to a fully connected layer, and the weight update to be computed as an outer-product of the neuron activations and the back-propagated errors. In the figure on the right, we illustrate how n^2 input vectors of dimension dk^2 (corresponding to the sub-regions of the image being convolved) are applied one-by-one as signals to the word lines of the array and the outputs representing the desired products are measured along the m bit-lines. These output vectors are rearranged to get the results of convolution operation.

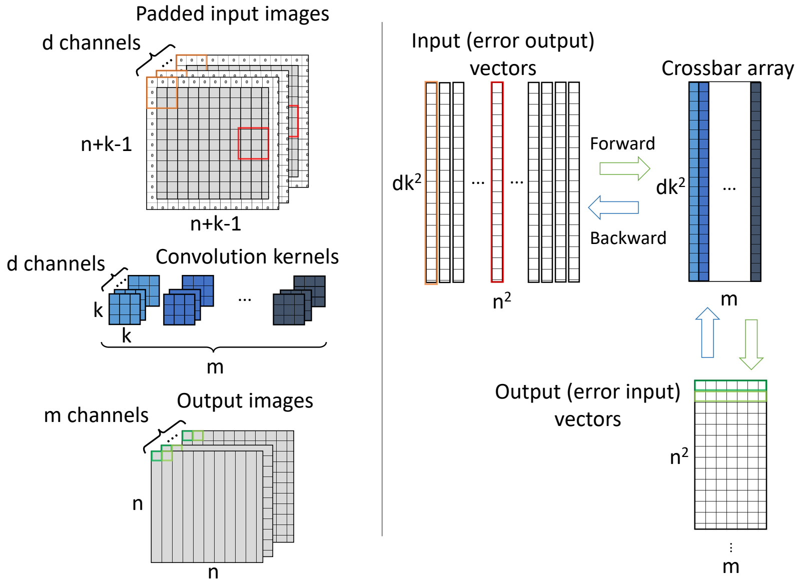

Supplementary Fig. 13. **Mapping 2D convolutions onto a memristive crossbar arrays** A 2D convolution can be represented as a matrix-matrix multiplication with the one matrix containing the image batches and the other the stretched convolution kernels. The filters are stretched out to 1D arrays and horizontally stacked on the memristive crossbar. The different image patches are extracted from the input image, stretched out and finally rearranged to form the columns of a large matrix. The convolution can now be computed by doing the matrix-matrix multiplication of these two matrices and reordering the output correctly. During the image patch extraction process we pad the original image with zero pixels at the border, to ensure that the output of our convolution has the same image size as the input. Considering an input image of size $n \times n$ and d channels and m convolution kernels of size $k \times k$ the dimensions of the input matrix is $dk^2 \times n^2$ and the dimensions of the matrix on the crossbar array is $dk^2 \times m$. The convolution can therefore be performed in n^2 matrix-vector multiplication cycles.

The details of the CNN architecture we used for CIFAR-10 image classification and its training implementation in MCA are shown in Supplementary Table II[6]. We chose a CNN with 9 layers; 6 convolution layers and 3 fully-connected layers for the simulation study presented in the main article. A pooling layer is present after 2 consecutive convolution layers. We employ the commonly used dropout regularization technique after the pooling layers and in-between the fully-connected layers. Light data augmentation is employed in the form of random image flipping and random adjustments of brightness and contrast. The number of parameters in this network is roughly 1.5 million.

Long Short Term Memory

The recurrent neural network (RNN) we used for the character level language modeling has two Long Short Term Memory (LSTM) units followed by a fully connected layer. The list of hyperparameters used in the training simulations can be found in Supplementary Table III. The simulation code is based on Chen Liang's TensorFlow implementation[7]. In this section, we discuss how the network is mapped to computational memory arrays in the MCA. LSTM units themselves are recurrent networks which when unrolled in time share weights among multiple time steps. The network topology of the LSTM cell in each time

Convolution 48 filters @ 3x3, ReLU
Convolution 48 filters @ 3x3, ReLU
Max Pooling, size 3x3, strides=2
Dropout, probability of dropping=0.25
Convolution 96 filters @ 3x3, ReLU
Convolution 96 filters @ 3x3, ReLU
Max Pooling, size 3x3, strides=2
Dropout, probability of dropping=0.25
Convolution 192 filters @ 3x3, ReLU
Convolution 192 filters @ 3x3, ReLU
Max Pooling, size 3x3, strides=2
Dropout, probability of dropping=0.25
Fully-Connected 1728x512, ReLU
Dropout, probability of dropping=0.5
Fully-Connected 512x256, ReLU
Dropout, probability of dropping=0.5
Fully-Connected 256x10, ReLU
Softmax

Optimization algorithm	SGD
Number of epochs	500
Learning rate	0.03, dropped by a factor of 0.1 at epochs 200
Batch size	100
Conductance refresh frequency	Every 51 batches

Supplementary Table II. Network structure and hyperparameters of the CNN for CIFAR-10 image classification.

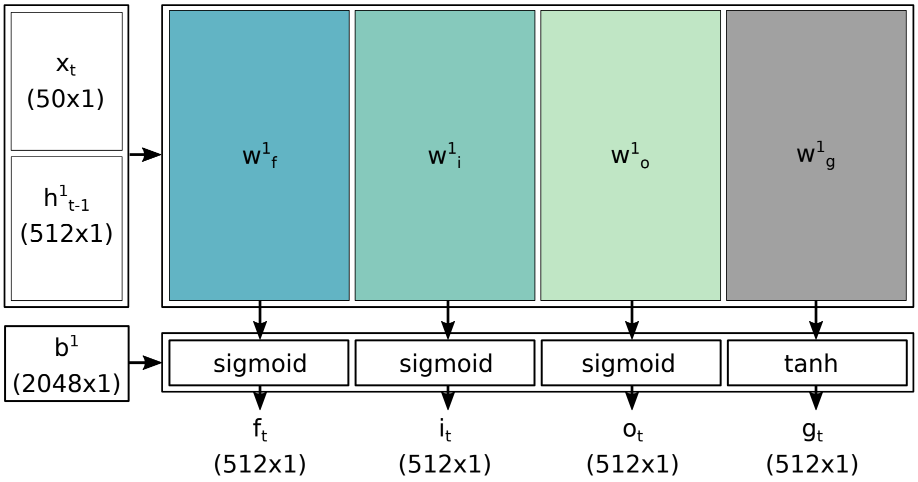

Supplementary Fig. 14. The mapping of the weights from the LSTM cell in the layer 1 to a crossbar array. Each colored square represents a fully-connected crossbar array of weights realized using memristive devices. The activation functions and the remaining gating operations (Equations (7) and (8)) are performed in the digital unit of the MCA.

step can be represented as a combination of multiple fully-connected layers. Hence, all the matrix-vector multiplications in the LSTM cell can be computed using crossbar arrays of memristive devices in a single cycle for each time step[8].

Optimization algorithm	SGD
Number of epochs	100
Learning rate	2.0, dropping by factor 0.25 at epochs 40 and 80
Batch size	32
Number of backpropagation-through-time steps	100
Dropout rate	0.15
Refresh frequency	Every 11 batches

Supplementary Table III. Hyperparameters used for the LSTM network training.

Each LSTM cell of layer l at time t has a cell state c_t^l that carries information through time steps. The cell operates on its hidden state from the previous time step (h_{t-1}^l) and on the training input (x_t) or hidden state from the previous layer (h_t^{l-1}) to generate intermediate states i_t (via input gate layer with weights w_i^l and *sigmoid* activation), f_t (via forget gate layer with weights w_f^l and *sigmoid* activation), o_t (via output gate layer with weights w_o^l and *sigmoid* activation), and g_t (via cell memory layer with weights w_g^l and *tanh* activation). These intermediate states are used to update the cell state and the hidden state using the

following relations:

$$\begin{pmatrix} i_t \\ f_t \\ o_t \\ g_t \end{pmatrix} = \begin{pmatrix} \sigma \\ \sigma \\ \sigma \\ tanh \end{pmatrix} \left[W \begin{pmatrix} x_t \text{ or } h_t^{l-1} \\ h_{t-1}^l \end{pmatrix} + b^l \right] \tag{6}$$

$$c_t^l = f_t \odot c_{t-1}^l + i_t \odot g_t \tag{7}$$

$$h_t^l = o_t \odot tanh(c_t^l) \tag{8}$$

where *sigmoid* (σ) and *tanh* are applied element-wise, $\odot$ is the element-wise multiplication, and b^l is a bias vector. W is obtained by stacking w_f^l, w_i^l, w_o^l, w_g^l as illustrated by Gokmen et al[8]. Here, x_t is of dimension $m = 50$ and i_t, f_t, o_t, g_t, h_t^l, and c_t^l are of dimension $n = 512$. Hence, the weight matrix W is of dimension $4n \times (n+m)$ in the first layer and $4n \times 2n$ in the second layer. For example, the mapping of weights in the first layer LSTM cell to the computational memory array is shown in Supplementary Fig. 14. Each of the weights matrices w_f^1, w_i^1, w_o^1, w_g^1 represents a fully-connected network of (512+50) inputs and 512 outputs. Since these four networks accepts the same input vectors, their weights can be mapped adjacent to each other in a fully-connected crossbar array with (512+50) common inputs and 2048 outputs. Dropout regularization is applied to the outputs of the two LSTM cells[9]. The 512 outputs of the second LSTM is connected to 50 softmax activation units to generate the final output via a fully-connected layer. The fully connected layer can be directly mapped to a crossbar array.

Generative Adversarial Network

A generative adversarial network (GAN) consists of two different neural networks, namely a generator and a discriminator. The generator generates images from purely random noise with some well-behaved distribution. The discriminator trains on images from both the generator and the training datasets and learns to classify whether the input image is from the training dataset or not. The objective of the generator network is to generate data that very closely resembles those from the training dataset and the objective of the discriminator is to distinguish between the generated and the dataset images.

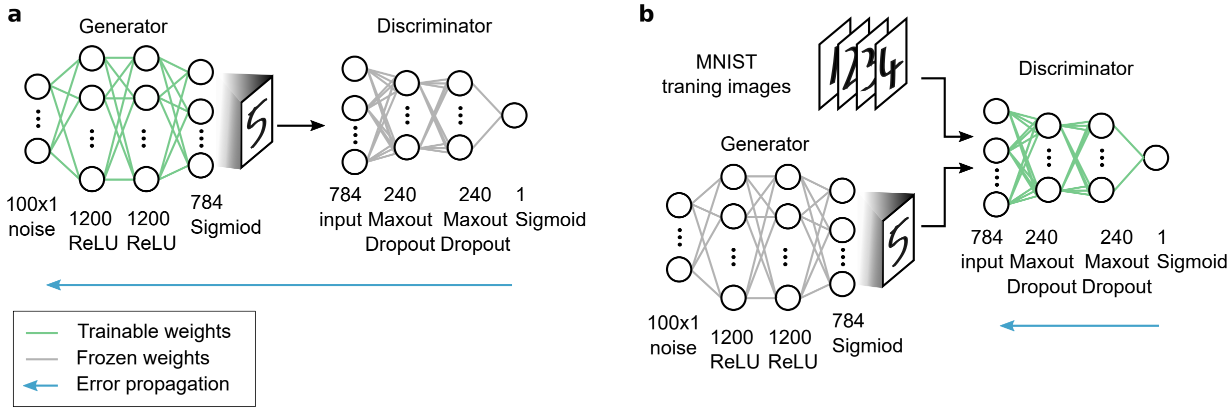

Supplementary Fig. 15. **Training process of the generator and discriminator network.** (a) Generator training. The discriminator weights are kept constant. The final error is computed at the output of the discriminator and it is back-propagated till the first weight layer of the generator (b) Discriminator training. The generator weights are held constant. The errors are back-propagated only for the discriminator weight layers.

Optimization algorithm	SGD with momentum of 0.5
Number of epochs	550
Learning rate	0.1, exponential learning rate decay with a decay rate of 0.996 after every epoch until it reaches 10^{-6}.
Batch size	100
Refresh frequency	Every 11 batches

Supplementary Table IV. Hyperparameters used for the training of the GAN.

Generator and discriminator are trained simultaneously on the training data. While training the generator, discriminator parameters are frozen and vice versa (See Supplementary Fig. 15). All the weights in the generator and the discriminator are initialized with normal distribution with zero mean and a standard deviation of 0.025. In the training of the GAN, the discriminator is trained once on a batch of 100 training images and 100 generated images. The generator is trained 2 times in MCA, and 4 times in FP32 training, on the same batch of 100 random noise inputs. The weights are updated after each batch. For the training, we used a momentum based stochastic gradient descent optimizer with a learning rate of 0.1 and a momentum of 0.5 for both networks. We used an exponential learning rate decay after every epoch with a decay rate of 0.996 until it reaches 10^{-6} and the momentum is kept constant. All the training images are normalized between $[0, 1]$ and no other preprocessing is performed on the images. Activations of the first two layers of the discriminator are scaled by factors of 1.25 and 2.00, respectively.

SUPPLEMENTARY NOTE 8: EFFICIENCY GAIN OF MCA WITH RESPECT TO 32-BIT AND MIXED-PRECISION FIXED POINT DIGITAL DESIGN

This section details the designs used to evaluate the computational efficiency of the MCA with respect to a 32-bit fully-digital design and a mixed-precision fully-digital design. The designs are optimized to train a two layer multi-layer perceptron (MLP) for handwritten digit classification similar to our training experiment in the main article (784 inputs, 250 hidden neurons, and 10 output neurons). First, we present an application specific integrated circuit (ASIC) design of the fully-digital high-precision (32-bit fixed point) training architecture implemented in 14nm low power plus (14LPP) technology. We evaluate its training time and energy based on post place and route results. Then, we discuss the computational memory implementation, energy consumption, and computing time. The design and analysis of the digital unit of the MCA in 14LPP are presented next. Finally, we summarize the results of the estimated energy and time to process one training example with the two designs.

32-bit digital ASIC design

In this section, we discuss the 32-bit fixed-point design for training and provide the power and throughput estimates. The ASIC design is optimized for the two layer MLP performing MNIST image classification and serves as a baseline for the corresponding MCA implementation. A block diagram of the architecture is presented in Supplementary Fig. 16.

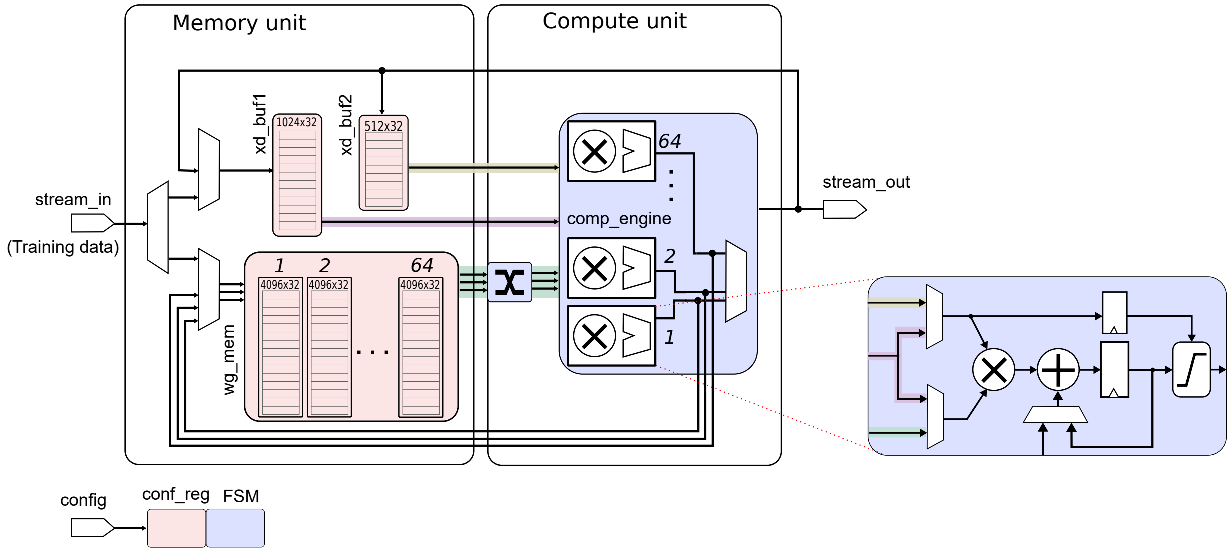

Supplementary Fig. 16. Hardware architecture of 32-bit fixed-point training design. Micro-architecture of one of the 64 parallel compute units is also shown.

Architecture

The 32-bit architecture consists of memory units, processing units, and configuration registers (*conf_reg*), in addition to a finite state machine (*FSM*) to control the training process. All the necessary memory is implemented using on-chip SRAM avoiding any off-chip communication. The on-chip storage can be divided into three sections. The area labeled *wg_mem* holds the weight values from the two weight layers. The areas labeled *xd_buf*1 and *xd_buf*2 are reserved for saving the activation values and gradients computed during the forward and backward propagation to use later for the weight update. The depth of the SRAM instance in *xd_buf*1 is set at 1024 and *xd_buf*2 is set at 512 considering the activation and gradient data vector sizes in the MLP. Further, the dataflow is designed such that for the data-propagation through the weight layers, one of the buffers from *xd_buf*1 and *xd_buf*2 serves as source of data while the other functions as the destination for results and they are interchanged for alternate layers. This permitted splitting storage into two single port SRAMs which are more area and energy efficient compared to using an equivalent dual-port SRAM.

There are 64 processing units operating in parallel. Correspondingly, there are 64 SRAM units in *wg_mem* such that they can be accessed in parallel and the processing units are maximally occupied during computations. During forward and backward propagation, 64 processing units located in *comp_engine* compute the dot product between an input vector and an array of 64

rows or columns of the weight matrix using multiply accumulate units over as many as clock cycles as the length of the input vector. Input vector is serially drawn from one of the xd_buf sites and fanned-out to all compute units while the elements from 64 streams of weight matrix values are drawn in parallel from the SRAM banks in wg_mem. The dot product results are subjected to ReLU activation or multiplied by gradient of ReLU function depending on whether it is a forward or a backward pass. The resulting elements of the output vector are stored in complementary xd_buf buffer. The above process is repeated until all the elements in the output vector are computed. The next layer propagation can be computed by feeding the last output vector as the input and writing the new output vector to its complementary xd_buf site. This routine is continued until propagations through all layers are completed.

The error at the final layer is computed using the last layer forward propagation activation data from one of the xd_bufs and the pre-stored labels from the other. For this network we use L2SVM[10] as the objective function to compute the gradient. During weight update, one of the xd_buf sites will first serially load 64 or less error gradients and then the corresponding activation vector is sent from the complementary xd_buf site while weight streams are drawn from wg_mem similar to forward propagation. The only difference is that the results from the compute units are written directly back to wg_mem in the next cycle to replace the old content. Therefore the read operations are throttled back at half the rate as forward propagation, to accommodate interleaved writes to SRAM.

One of the 64 parallel compute units comprises of a 32-bit multiplier, a 32-bit adder, a register and a post processing unit that perform clipping and binary multiply operations, in addition to some multiplexers. The proposed micro-architecture supports compute operations visible in all stages of training of the MLP. Depending on the subtask to be executed, appropriate values can be applied on the multiplexer and the post processing unit to configure the compute core accordingly. Transitions from subtasks is controlled by the FSM, that can be configured with DNN hyper-parameters by an external interface. We adapt a 32-bit fixed point number format with 23-bit fractional part, 8-bit integer part, and 1 sign bit for the datapath. Therefore, all the SRAM instances have 32-bit word size.

Implementation

The 32-bit training hardware architecture is synthesized using Samsung 14nm technology files. The post route netlist reports a maximum operating frequency of 680 MHz at 0.72V and an area of 1.26 mm^2 which is equivalent to 5.21 million gates when measured with respect to the smallest 2-input NAND gate in the 14nm library. When operating at 500 MHz frequency, the design achieves a throughput of 43k images per second while dissipating 0.62 W power on average.

MCA – Computational memory unit

Here, we detail the design and energy consumption of the PCM based computational memory. In addition to the PCM crossbar array, all the peripheral circuits for data transfer, translation between the analog and digital domain, and for programming the PCM devices are taken into account, allowing an in-depth comparison to a functionally equal digital system in terms of speed and energy consumption.

Supplementary Table V. Summary of PCM crossbar array specifications

Parameter	Name	Value
	Technology	14 nm
$(n_{rows,1}, n_{cols,1})$	Number of input and output neurons in layer 1	(785, 250)
$(n_{rows,2}, n_{cols,2})$	Number of input and output neurons in layer 2	(251, 10)
n_{PCM}	Number of PCM devices per synapse	2 (G_p and G_n)
G_{PCM}	PCM device conductance range	$100\,nS - 10\,\mu S$
V_{read}	Read voltage	100 mV
V_{DD}	Digital supply voltage	0.8 V
	Input application method	Pulse Width Modulation (PWM)
f_{clk}	Clock frequency	2 GHz
$n_{inputbits}$	Resolution of the PWM inputs	8 bit
$n_{outputbits}$	ADC output resolution	8 bit

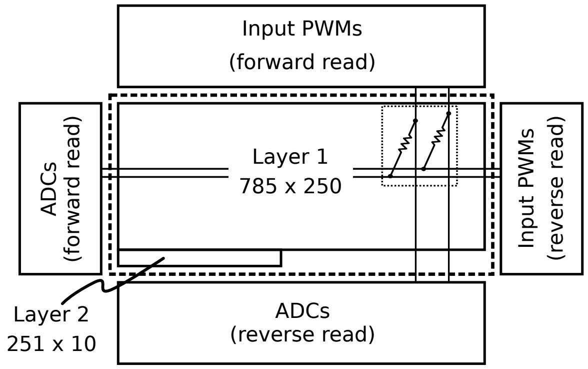

Supplementary Fig. 17. Top level system diagram of computational memory unit. The crossbar array has 785 input neurons and 260 output neurons. Each synapse between the neurons is realized using two PCM devices in differential configuration. The PCM array has 785 × 520 devices in total. It accommodates two weight layers of dimensions 785 × 250 and 251 × 10. Two sets of pulse width modulators (PWMs) and analog to digital converters (ADCs) permit data propagation in forward and backward (reverse) direction.

The system specifications considered in this analysis are listed in Supplementary Table V. A crossbar containing 785 inputs and 260 outputs is assumed, so that both layers of the 784-250-10 MNIST network can be accommodated. The two weight layers of the network are positioned side by side (as shown in Supplementary Fig. 17) and hence have to be read separately during forward or backward propagation stages. Each synapse consists of two doped-$Ge_2Sb_2Te_5$ based mushroom-type PCM cells, G_p and G_n, in a differential configuration[11]. An additional input row per layer is added to accommodate the bias term implemented using the PCM devices. We employ pulse width modulation (PWM) circuits to convert digital numbers to analog read voltage pulses and use analog to digital converters (ADCs) to read the array outputs. Using two sets of PWMs and ADCs we can perform the read operation on both forward and backward propagation cycles.

Energy consumption during read

Different processes involved in the analog computation are shown in Supplementary Fig. 18. In the following paragraphs, the functionality and implementation of each process will be explained and numbers for energy consumption will be given. It will be assumed that all PCM related programming procedures have concluded prior to readout.

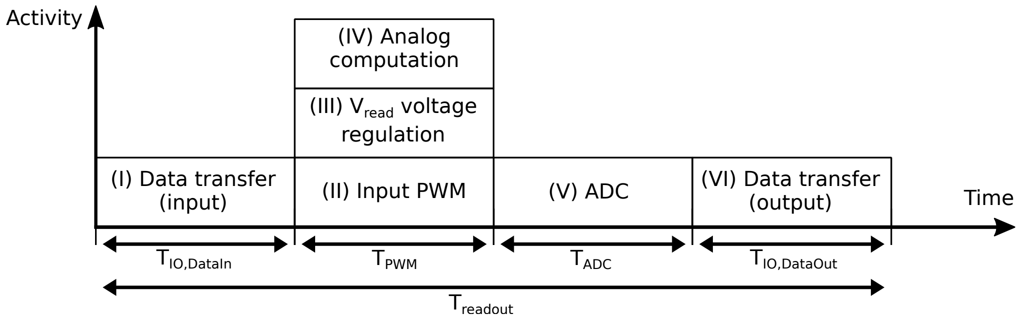

Supplementary Fig. 18. Processes during readout (matrix-vector multiplication) procedures in the computational memory unit.

(I) **Data transfer (input)**
The readout procedure starts with a data transfer operation of the input vector for the matrix-vector multiplication to the registers of the PWM modulator. For this, a 16-bit wide input bus and a 1-bit latch enable signal is used. Therefore, the data transfer energy can be obtained using the energy required per bit shift operation, $E_{bitshift} = 2\,\text{fJ}$ obtained from circuit simulations, inserted in the following equation:

$$E_{I,DataIn} = E_{bitshift} \times n_{rows,Layer} \times (n_{bits,PWM} + n_{rows,Layer}/4) \tag{9}$$

A number of $n_{rows,Layer}/2$ cycles is needed for all data to be transmitted, since the bus width matches two 8-bit input data.

$$T_{IO,DataIn} = n_{rows,Layer} \times \frac{1}{2 \times f_{clk}} \tag{10}$$

(II) **Input Pulse Width Modulation**

After the input vector has been transmitted to the dedicated registers, it is applied by means of PWM to the analog array. The modulator effectively performs a Digital-to-Analog conversion from an 8 bit signed number to a read voltage pulse. In this study the PWM circuit block is assumed to comprise of a central counter unit and as many digital comparators blocks as there are rows (or columns for reverse read) in the crossbar. Using the average energy per counting step $E_{bit,counter} = 50\,\text{fJ}$, which was obtained from simulations, one can calculate the energy portion necessary to generate the digital ramp signal for the modulator. The capacitive load driven by the counter outputs, which consists of $n_{rows,Layer} \times n_{inputbits}$ comparator inputs was also taken into account, although its contribution is minimal (A C_{load} of $100\,\text{aF}$ per comparator input was determined from circuit simulation). The comparators are implemented as combinatorial logic. Their respective outputs, which enable and disable the PWM pulses, are furthermore buffered up to assure signal propagation at negligible delay. This is done at a cost of $E_{PWM,Buffer} = 10\,\text{fJ}$ per turn-on and turn-off event. By adding the counter energy $E_{PWM,counter}$, load related energy $E_{PWM,load}$ and comparator energy $E_{PWM,comparator}$, the overall PWM energy contribution is obtained:

$$E_{PWM,counter} = 2^{n_{inputbits}} \times E_{bit,counter} \tag{11.1}$$

$$E_{PWM,load} = n_{rows,Layer} \times \frac{1}{2} \times C_{load} \times V_{DD}^2 \times \sum_{x=1}^{n_{inputbits}} 2^x \tag{11.2}$$

$$E_{PWM,comparator} = 2 \times n_{rows,Layer} \times E_{PWM,Buffer} \tag{11.3}$$

$$E_{II,PWM} = E_{PWM,counter} + E_{PWM,load} + E_{PWM,comparator} \tag{11.4}$$

The PWM period consists of the time that is necessary for the counter to count up to $2^{n_{inputbits}}$.

$$T_{PWM} = 2^{n_{inputbits}} \times \frac{1}{f_{clk}} \tag{12}$$

(III) **Read voltage regulation**

With the inputs being applied on the crossbar rows via PWM, the columns of the crossbar are kept at a constant potential, so that the voltage across the PCM devices corresponds to the read voltage V_{read}. This voltage regulation is achieved through the usage of Operational-Transconductance-Amplifiers (OTAs). Using the standby bias current $I_{OTA} = 50\,\mu\text{A}$ and assuming the on-time to be matched to the PWM period T_{PWM} the energy spent on operational amplifiers can be determined as:

$$E_{III,OTA} = n_{columns,Layer} \times V_{DD} \times I_{OTA} \times T_{PWM} \tag{13}$$

(IV) **Analog computation**

The energy consumption of the computation being performed in the analog domain is determined with the following assumptions: First, all conductance values are set to the average conductance observed from the training experiment of $G_{PCM,avg} = 2.32\,\mu\text{S}$. This applies to both the positive and negative conductances G_p and G_n ($n_{PCM} = 2$) in a differential PCM implementation. Second, all the inputs are set to an average by using half the maximum duration of the PWM read voltage pulse T_{PWM}. Third, it is assumed that the analog computation current is sourced from the main supply voltage V_{DD}. Finally the contribution of the read current mirrored to the ADCs is ignored, due to the down mirroring ratio of around $\frac{1}{1000}$. The total analog computation energy is:

$$E_{IV,PCM} = n_{PCM} \times V_{DD} \times (V_{read} G_{PCM,avg}) \times n_{rows,Layer} \times n_{columns,Layer} \times \frac{1}{2} T_{PWM} \tag{14}$$

(V) **Analog to Digital Converters (ADCs)**

The power and speed figures of an ADC circuit[12] designed in the same technology were used as a basis for obtaining the energy consumed for converting the analog voltages to digital quantities. One analog to digital conversion is shown to require an energy of $E_{A-to-D} = 3\,\text{pJ}$ and a time of $t_{A-to-D} = 1\,\text{ns}$. The total number of conversions corresponds to the number of active columns $n_{columns,Layer}$ in the crossbar, which in turn depends on the layer size. It is assumed that one ADC is shared among 4 columns or rows in the crossbar. Powering on the ADC requires a turn-on time of $t_{ADC,on}$ equal to two conversion cycles.

$$E_{V,ADC} = n_{columns,Layer} \times E_{A-to-D} \tag{15}$$

$$T_{ADC} = t_{ADC,on} + 4 \times t_{A-to-D} = 6 \times t_{A-to-D} \tag{16}$$

(VI) **Data transfer (Output)**
Finally, all the data generated by the ADCs, which represent the result vector of the analog matrix-vector multiplication, is sent out using a 16 bit wide bus.

$$E_{VI,DataOut} = E_{bitshift} \times n_{columns,Layer} \times (n_{bits,ADC} + n_{columns,Layer}/4) \tag{17}$$

$$T_{IO,DataOut} = n_{columns,Layer} \times \frac{1}{2\times f_{clk}} \tag{18}$$

Supplementary Table VI. Summary of readout energy consumption and time per layer per training image

Parameter	Layer 1 - forward read	Layer 2 - forward read	Layer 2 - reverse read
$n_{rows,Layer}$	785	251	10
$n_{columns,Layer}$	250	10	251
$E_{I,DataIn}$	0.320 nJ	0.035 nJ	0.00021 nJ
$E_{II,PWM}$	0.041 nJ	0.022 nJ	0.013 nJ
$E_{III,OTA}$	1.28 nJ	0.051 nJ	1.285 nJ
$E_{IV,PCM}$	4.66 nJ	0.060 nJ	0.060 nJ
$E_{V,ADC}$	0.750 nJ	0.030 nJ	0.753 nJ
$E_{VI,DataOut}$	0.0353 nJ	0.00021 pJ	0.0353 nJ
E_{total}	7.089 nJ	0.198 nJ	2.146 nJ
$T_{readout}$	392.75 ns	199.25 ns	199.25 ns

Energy consumption during programming

The main contribution in the energy consumption during programming the PCM devices, for the synaptic weight update, is from the analog current mirrors applying the programming pulse on the selected PCM device. Contributions from the logic circuits have been found to be negligible, as only minimal amounts of data needs to be transmitted to select the PCM device to be programmed and together with the decoder and internal timing circuits, an energy less than 100 fJ is required. Input/output (IO) and address decoding has been found to require around $T_{prog,logic} = 10\,\text{ns}$. The programming current of $I_{prog} = 90\,\mu\text{A}$ will be provided over a PMOS current mirror, which is connected to a 3.2 V supply and which needs a settling time of $T_{prog,settle} = 10\,\text{ns}$ in addition to the required pulse width of $T_{prog,pulse} = 50\,\text{ns}$. Due to the mirror, the current is sourced twice which adds a factor of two to the energy equation:

$$E_{prog,sparse} = 2 \times I_{prog} \times 3.2\,\text{V} \times (T_{prog,settle} + T_{prog,pulse}) = 34.5\,\text{pJ} \tag{19}$$

$$T_{prog,sparse} = T_{prog,logic} + T_{prog,settle} + T_{prog,pulse} = 70\,\text{ns} \tag{20}$$

In order to perform a refresh operation for the PCM devices whose conductance are saturated due to the repeated SET operation, we need to read and RESET the PCM devices based on the refresh condition. The energy for one device read, E_{read}, involves input PWM, read voltage regulation, analog read, and ADC. The read pulse duration is assumed to be one fourth of the T_{PWM}.

$$E_{read} = 2^6 \times E_{bit,counter} + E_{PWM,load} + V_{DD} \times I_{OTA} \times \tfrac{1}{4}T_{PWM} + V_{DD} \times (\tfrac{1}{2}V_{read}G_{PCM,max}) \times \tfrac{1}{4}T_{PWM} + E_{A-to-D} = 20.3\,\text{pJ} \tag{21}$$

$$T_{read} = T_{ADC} + \tfrac{1}{4}T_{PWM} = 35\,\text{ns} \tag{22}$$

The RESET is performed by connecting the PCM cell (PCM plus access device) to a 3.2 V supply line for a duration T_{reset} of 50 ns. The energy consumption during RESET, E_{reset}, is computed assuming a RESET current I_{reset} of 360 μA.

$$E_{reset} = 3.2\,V \times I_{reset} \times T_{reset} = 57.6\,\text{pJ} \tag{23}$$

MCA – Digital unit ASIC design

In this section, hardware design of the digital unit in the MCA to train the two-layer MLP is discussed. The digital subsystem coordinates the forward and backward propagation of data through the computational memory, and performs the remaining computations and weight update accumulation in order to complete the training procedure.

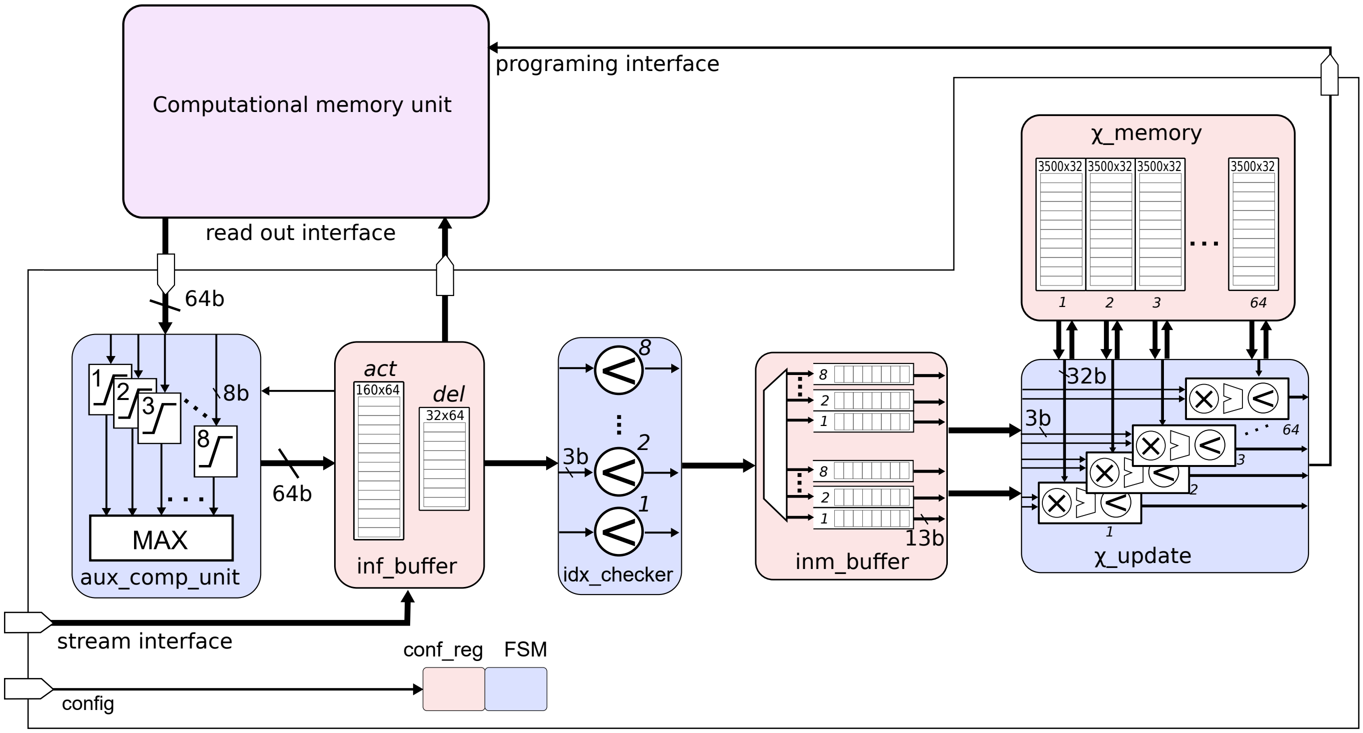

Supplementary Fig. 19. The hardware architecture of the digital unit of the MCA. The interaction of the digital unit with the computational memory unit is also shown.

Architecture

Supplementary Fig. 19 illustrates the computational blocks in the ASIC design. It consists of necessary processing circuits and on-chip memory for buffering intermediate results and weight update accumulation. An auxiliary compute unit (*aux_comp_unit*) implements ReLU, ReLU-gradient, and L2SVM based error computation at the final layer using the results from the computational memory. The *aux_comp_unit* also determines the maximum of the backpropagated error vector which is used later to scale the normalized weight update in the χ*_update* unit. The *inf_buffer* stores the activations and error gradients computed and the input to the first layer streamed externally. The *inf_buffer* stores data in 8-bit format; at the precision of the ADCs at the computational memory periphery. At the operating frequency of 500 MHz of the digital unit, a maximum of 8 data tokens are supplied or admitted by the crossbar at a time, and hence the *inf_buffer* hosts an SRAM of word size of 64, to accommodate storing/retrieving eight 8-bit values in a single cycle. The SRAM has a depth of 192 entries in total to support the data from 2-layer MLP for MNIST classification.

Weight update process starts as soon as the backward propagation results are stored in the *inf_buffer* for one layer. As the first step, error gradient data of the layer, and activation data of the prior layer are quantized to 3-bit and forwarded to *idx_checker* module, 8 tokens per cycle. As the majority of quantized data are zero, they have no contribution to weight update. The *idx_checker* determines the indices of non-zero entries from the post quantized data and stores them in *inm_buffer*. *inm_buffer* is a pool of queues and eight queues are assigned for each activation and error gradient data. The queues exploit maximum operational capacity of χ*_update* unit for the longest possible time by uniformly spreading non-zero activation and error gradient data into the 8 different queue lanes. The queues feature a special *read_reset* input that returns the read pointer of the queue to a predefined location. This enables iterating through the entire non-zero error gradient list multiple rounds, once for each of the 8 non-zero activation values set. The χ*_update* unit has 64 parallel compute units which performs the 3-bit multiplication, scale the results using bit-shifts and accumulate them over the 32-bit numbers from the χ*_memory*. It also verifies if the accumulation

exceed a pre-programmed threshold and outputs the addresses of the PCM devices to be programmed in the computational memory. The *χ_memory* are organized as 64 SRAM modules to enable parallel access for the compute units. The queues in *inm_buffer* are filled in 172 cycles and 64 entries in the *χ_memory* are updated every two cycles. The length of the weight update stage depends on the number of non-zero entries in the queues, and the average number of non-zero entries is observed to be below 1% of the total number of the synapses in the MLP for the 3-bit outer product implementation.

Implementation

The digital unit of MCA design for the MLP is synthesized using Samsung 14nm technology files. The post route netlist achieves a maximum frequency of 510 MHz at 0.72 V operating voltage. The digital unit has a total area of 0.92 mm^2, an equivalent of 3.83 million NAND gates. The digital unit was clocked at 500 MHz for the energy estimations which in combination with the computational memory unit enables MCA to achieve a maximum processing rate of 495k images per second, while dissipating 41.27 mW power on average.

Fully digital mixed-precision ASIC design

In this section, the hardware design of the fully digital mixed-precision architecture to train the two-layer MLP is discussed. The block diagram of the architecture is presented in Supplementary Fig. 20.

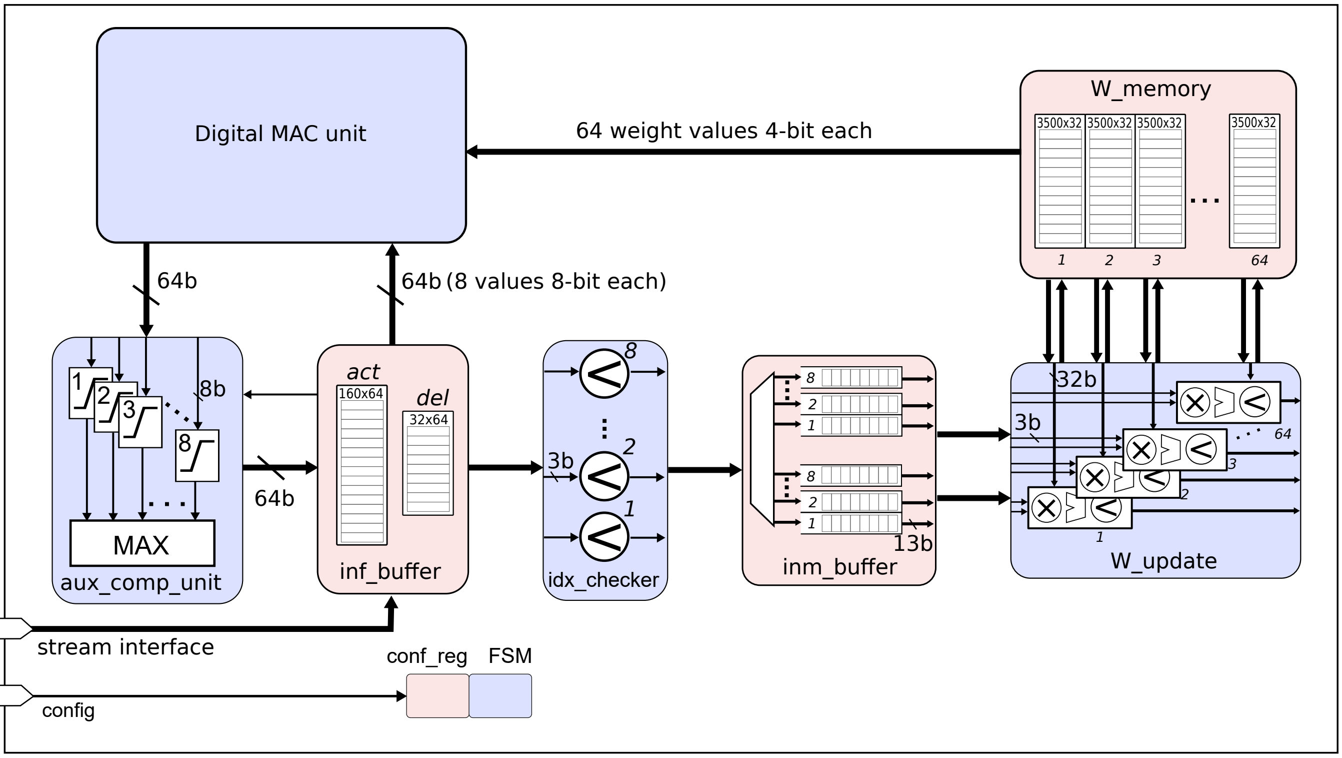

Supplementary Fig. 20. The hardware architecture of the fully digital mixed-precision designs.

Architecture

The architecture of the digital mixed-precision design is similar to that from the MCA, except that the multiply-accumulate operation during the data propagation stages are performed using a digital MAC unit instead of a computational memory unit. The weights are stored in 32-bit precision. The Digital MAC unit uses 4-bit version of the weight values from *W_memory* and 8-bit activations/error vectors from *inf_buffer* to perform multiply-accumulate during data propagation stages. The Digital MAC unit has eight sub MAC units (Supplementary Fig. 21) each of which receive eight 4-bit weight values and eight 8-bit

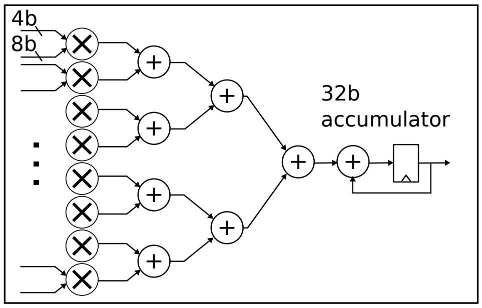

Supplementary Fig. 21. The micro-architecture for one of the eight sub MAC units in the fully digital mixed-precision design. One sub MAC unit implements eight multiplications and their accumulation at a time.

activation/error values. The operands are multiplied element wise and added together using an adder tree in a single cycle. The results from multiple cycles are accumulated in corresponding 32-bit accumulators to generate eight multiply-accumulate results. 8-bit truncated versions of the results are transferred to an auxiliary compute unit (*aux_comp_unit*). The *aux_comp_unit* implements ReLU, ReLU-gradient, and L2SVM based error computation at the final layer. The *aux_comp_unit* also determines the maximum of the backpropagated error vector which is used later to scale the normalized weight update in the *W_update* unit. The *aux_comp_unit* results are stored in *inf_buffer*.

The weight update stage follows the same architecture and the mixed-precision optimization as that from the MCA. The χ*_memory* and χ*_update* units from MCA are replaced by the *W_memory* and *W_update* units here since we are updating the actual high-precision version of the weight values and does not involve analog memory device programming. *idx_checker* module fills the queues in the *inm_buffer* using non-zero 3-bit quantized versions of the neuron activations and error values along with their corresponding indices. *W_update* unit performs the 3-bit multiplications of activations and errors, scale the results via bit shifts to represent the actual weight update magnitude, and the results are accumulated to the corresponding 32-bit weight values from the *W_memory*.

Implementation

The digital mixed-precision design for the MLP is synthesized using Samsung 14nm technology files. The post route netlist achieves a maximum frequency of 682 MHz at 0.72 V operating voltage and has a total area of 0.92 mm^2. The design was clocked at 500 MHz for the energy estimations like the previous two designs. It achieves a maximum processing rate of 136k images per second, while dissipating 0.25 W power on average.

Results

Supplementary Table VII summarizes the results of the energy and time used for the processing of one training example for the 32-bit, fully digital mixed-precision design and MCA designs. In this study, the energy and time for the initial data load from external memory is neglected in the designs. While the maximum frequencies of the 32-bit design, fully digital mixed-precision design and the digital unit of MCA designs were 680 MHz, 682 MHz and 510 MHz, respectively, all the designs were clocked at 500 MHz during energy estimation for the ease of comparison. The computational memory peripherals were clocked at 2 GHz and operate in sync with the corresponding digital unit of the MCA. The 32-bit design consumes 14.35 μJ per image and achieves a training throughput of 43k images per second. The fully digital mixed-precision design consumes 1.87 μJ per image and achieves a training throughput of 136k images per second. The energy estimate for the MCA architecture is obtained by combining the energy consumption from the computational memory and the digital unit. The data propagation through the two layers in the computational memory happens sequentially and the neuron activations and error vectors are stored in the allocated buffers in the digital unit. The $T_{readout}$ in the Supplementary Table VI is the time for performing the matrix-vector multiplication and the associated data transfer in the computational memory unit. The data transfer time (Equations (10) and (18)) for forward propagation through the layers 1 and 2 is 258.75 ns and 65.25 ns, respectively, and for backward propagation through layer 2 is 65.25 ns. Since this time for data transfer between computational memory unit and the digital unit is already included in the time estimate of the digital unit, they are subtracted from the $T_{readout}$ to determine the time for computational memory in Supplementary Table VII. The energy consumption of the weight update stage in MCA was computed by feeding data vectors which resulted in 2130 ($\sim$1% of total synapse count) non-zero weight updates. The weight update stage determines the addresses of the PCM devices to be programmed and places them in a queue. The PCM programming in the regular weight

update and the conductance refresh for the differential configuration happen in parallel with the weight update computation in the digital unit. After each device update, the conductance pair (G_p and G_n) for the synapse is read and if the refresh condition is met, both devices are RESET and the conductance difference is programmed to one of the devices using SET pulses.

We used the statistics of device programming from the training experiment to determine the PCM programming energy for the MCA design. On average, one PCM synapse received a regular device update (implying 1 SET pulse and 2 device conductance reads) every 2 training examples, and one PCM synapse was refreshed (implying 2 RESET pulses and 3 SET pulses on average) every 18 training examples. Including the energy for IO and address decoding, device programming (consisting of prorated regular device update and refresh) consumes 49.62 pJ on average per image. Raw update time per PCM synapse (without refresh) is 140 ns, which is much shorter than the weight update stage in the digital unit. Since PCM programming happens in parallel to the weight update computation in the digital unit, it does not incur additional time overhead and therefore weight update time of computational memory is not included in Supplementary Table VII. Together, the MCA design consumes 83.2 nJ per image at a throughout of 495k training images per second.

Finally, in Supplementary Fig. 22, we show that the implementation of the digital unit of the MCA gives a test accuracy comparable to our experimental results for MNIST image classification.

Supplementary Table VII. Energy and time estimated based on application specific integrated circuit (ASIC) designs for processing one training image in MCA and corresponding fully digital 32-bit and mixed-precision designs. The numbers are for a specific two layer perceptron with 785 input neurons, 250 hidden neurons, and 10 output neurons.

Architecture	Parameter	Forward propagation	Backward propagation	Weight update	Total
32-bit design	Energy	5.62 μJ	0.09 μJ	8.64 μJ	14.35 μJ
	Time	7.31 μs	0.59 μs	15.36 μs	23.27 μs
Fully digital mixed-precision design	Energy	1.78 μJ	0.016 μJ	0.076 μJ	1.87 μJ
4-bit weights, 8-bit activations/errors	Time	6.41 μs	0.13 μs	0.79 μs	7.33 μs
MCA – computational memory	Energy	7.29 nJ	2.15 nJ	0.05 nJ	
	Time	0.27 μs	0.13 μs	–	
MCA – digital unit	Energy	8.97 nJ	2.76 nJ	61.98 nJ	
	Time	0.34 μs	0.09 μs	1.19 μs	
MCA – total	Energy	16.3 nJ	4.91 nJ	62.03 nJ	83.2 nJ
	Time	0.61 μs	0.22 μs	1.19 μs	2.02 μs

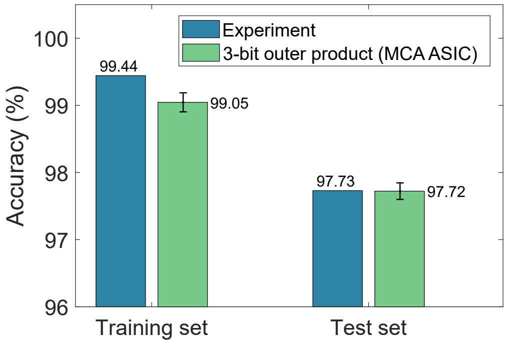

Supplementary Fig. 22. The training and test accuracies of the MCA ASIC design using modeled PCM devices in contrast to the training experiment presented in Section III of the main article. In the ASIC hardware design, we used ReLU activation functions for the hidden layer neurons and L2SVM to compute the output layer errors. The neuron activations and backpropagated errors are converted to signed 3-bit numbers by scaling and rounding. The scaling factors are chosen to be powers of two such that the hardware operations can be implemented as bit-shifts. The weight updates are computed as outer-product of these 3-bit numbers and the resulting sparse matrix is multiplied using the learning rate and scaling factors which again are powers of two. We used a mini-batch size of 1 and learning rate of 2^{-8}. The network was trained using the MCA for 40 epochs using the 60,000 training images and the classification performance was evaluated using the 10,000 test images from the MNIST dataset.

SUPPLEMENTARY REFERENCES

[1]I. Boybat, M. Le Gallo, S. Nandakumar, T. Moraitis, T. Parnell, T. Tuma, B. Rajendran, Y. Leblebici, A. Sebastian, and E. Eleftheriou, "Neuromorphic computing with multi-memristive synapses," Nature communications **9**, 2514 (2018).

[2]S. Nandakumar, M. Le Gallo, I. Boybat, B. Rajendran, A. Sebastian, and E. Eleftheriou, "Mixed-precision training of deep neural networks using computational memory," arXiv preprint arXiv:1712.01192 (2017).

[3]N. Papandreou, H. Pozidis, A. Pantazi, A. Sebastian, M. Breitwisch, C. Lam, and E. Eleftheriou, "Programming algorithms for multilevel phase-change memory," in *IEEE International Symposium on Circuits and Systems (ISCAS)* (IEEE, 2011) pp. 329–332.

[4]A. Shafiee, A. Nag, N. Muralimanohar, R. Balasubramonian, J. P. Strachan, M. Hu, R. S. Williams, and V. Srikumar, "ISAAC: A convolutional neural network accelerator with in-situ analog arithmetic in crossbars," in *2016 ACM/IEEE 43rd Annual International Symposium on Computer Architecture (ISCA)* (2016) pp. 14–26.

[5]T. Gokmen, M. Onen, and W. Haensch, "Training Deep Convolutional Neural Networks with Resistive Cross-Point Devices," Frontiers in Neuroscience **11**, 1–22 (2017), 1705.08014.

[6]P. Kaur, "Convolutional neural networks (CNN) for CIFAR-10 dataset," `http://parneetk.github.io/blog/cnn-cifar10/` (2017).

[7]L. Chen, `https://github.com/crazydonkey200/tensorflow-char-rnn`.

[8]T. Gokmen, M. Rasch, and W. Haensch, "Training LSTM networks with resistive cross-point devices," Frontiers in neuroscience **12**, 745 (2018).

[9]W. Zaremba, I. Sutskever, and O. Vinyals, "Recurrent neural network regularization," arXiv preprint arXiv:1409.2329 (2014).

[10]Y. Tang, "Deep learning using linear support vector machines," (2013) arXiv:1306.0239.

[11]G. W. Burr, R. M. Shelby, S. Sidler, C. Di Nolfo, J. Jang, I. Boybat, R. S. Shenoy, P. Narayanan, K. Virwani, E. U. Giacometti, *et al.*, "Experimental demonstration and tolerancing of a large-scale neural network (165 000 synapses) using phase-change memory as the synaptic weight element," IEEE Transactions on Electron Devices **62**, 3498–3507 (2015).

[12]L. Kull, D. Luu, C. Menolfi, M. Braendli, P. A. Francese, T. Morf, M. Kossel, H. Yueksel, A. Cevrero, I. Ozkaya, and T. Toifl, "A 10b 1.5GS/s pipelined-SAR ADC with background second-stage common-mode regulation and offset calibration in 14nm CMOS FinFET," in *2017 IEEE International Solid-State Circuits Conference (ISSCC)*, pp. 474–475.
